# Supplementary material for: Integrated microwave photonic notch filter using a heterogeneously integrated Brillouin and active-silicon photonic circuit
Source: Nat Commun. 2023 Nov 20;14:7544. doi: 10.1038/s41467-023-43404-x (PMC10662262; doi:10.1038/s41467-023-43404-x)
Supplement: Supplementary file 1 — Supplementary Information [file 41467_2023_43404_MOESM1_ESM.pdf]

Supplementary Information:
"Integrated microwave photonic notch filter using a heterogeneously integrated Brillouin and active-silicon photonic circuit"

Matthew Garrett,[1,2] Yang Liu,[1,2] Moritz Merklein,[1,2,*] Cong Tinh Bui,[1,2] Choon Kong Lai,[1,2]
Duk-Yong Choi,[3] Stephen J. Madden,[3] Alvaro Casas-Bedoya,[1,2] and Benjamin J. Eggleton[1,2,†]

[1] *Institute of Photonics and Optical Science (IPOS), School of Physics, The University of Sydney, NSW, 2006, Australia*
[2] *The University of Sydney Nano Institute (Sydney Nano), The University of Sydney, NSW, 2006, Australia*
[3] *Laser Physics Centre, Department of Quantum Science and Technology, Research School of Physics, Australian National University, Canberra, ACT, 2601, Australia*

I. AS$_2$S$_3$ WAVEGUIDE LOSSES

The overall As_2S_3 waveguide losses (L_Σ) can be separated into two contributions. The first contribution is from the Si-As_2S_3 interface, and the second is from the propagation losses in the As_2S_3. We can therefore express the overall loss as

$$L_\Sigma = 2L_{\text{Si-As}_2\text{S}_3} + \alpha l_{\text{As}_2\text{S}_3} \tag{S1}$$

where $L_{\text{Si-As}_2\text{S}_3}$ is the loss per Si-As_2S_3 interface (dB), α is the propagation loss in the As_2S_3 waveguide (dB/cm) and $l_{\text{As}_2\text{S}_3}$ is the length of the As_2S_3 waveguide (cm).

We measured the overall optical losses directly by measuring the input and output optical power of the Si-As_2S_3 chip. A fibre array was used to couple light onto the Si-As_2S_3 chip. A pair of shunt silicon grating couplers allowed for the grating coupler losses to be measured and compensated for in the loss calculation of the As_2S_3 waveguides. Each grating coupler had a loss of ≈ 3.5 dB.

The waveguide propagation losses were extracted using a LUNA OVA5000 optical domain frequency reflectometer (OFDR) and fitting a line to the measured reflected signal. The experimental setup for the measurements of the overall optical losses and the propagation losses are shown in Fig

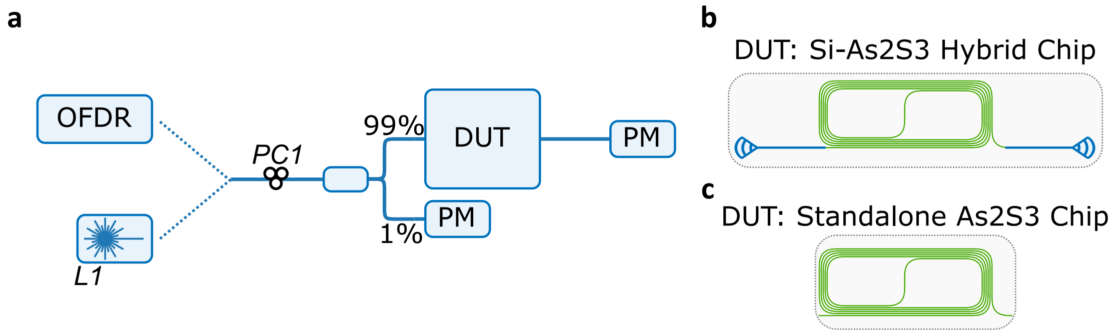

FIG. S1. **Experimental setup for loss measurements of the As_2S_3 waveguides.** (a) Setup for OFDR and total insertion loss measurements. (b) Simplified chip schematic of the Si-As_2S_3 chip. (c) Simplified schematic of standalone As_2S_3 chip. OFDR: Optical frequency domain reflectometry, L: Laser PC: Polarisation controller, PM: Power meter DUT: Device under test.

* moritz.merklein@sydney.edu.au

† benjamin.eggleton@sydney.edu.au

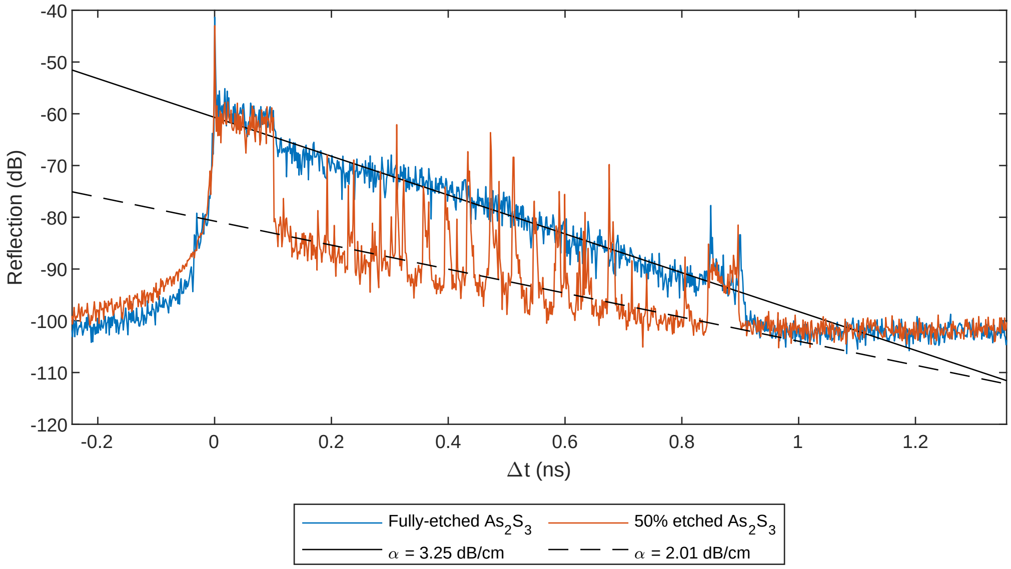

FIG. S2. **OFDR measurement made on on the Si-As_2S_3 platform.** The fully-etched (blue) and 50% etched (orange) waveguide losses are shown. The propagation losses are extracted from the linear fits.

S1 (a). A high-level schematic of the heterogeneous Si-As_2S_3 chip with silicon grating couplers is shown in Fig. S1 (b) and a schematic of the standalone As_2S_3 chip is shown in Fig. S1 (c).

A. As_2S_3 Propagation Loss Measurements

Fig. S2 shows the measured OFDR traces taken from the Si-As_2S_3 platform. The peak in reflection at $\Delta t = 0$ is due to the index mismatch between the fibre array and the silicon grating coupler. The section from $0 < \Delta t < 0.15$ ns is the reflection from the silicon waveguide. The region corresponding to $0.15 < \Delta t < 0.82$ ns is a reflection from the As_2S_3 waveguide. The reflection from the second Si-As_2S_3 interface is seen at $\Delta t = 0.82$ ns, and the reflection from the second grating coupler can be seen at $\Delta t = 0.9$ ns.

The blue trace in Fig. S2 corresponds to the measured signal in the fully-etched As_2S_3 waveguide. The solid line of fit indicates a propagation loss of 3.25 dB/cm. The orange trace represents the signal from the 50% etched rib waveguide. The dashed line of fit indicates a propagation loss of 2.01 dB/cm. The peaks in the rib waveguide measurement are due to localised scattering as the optical signal propagates through the As_2S_3 waveguide. This scattering is likely due to the contamination that occurred during post-processing. Furthermore, the difference in the reflected signal magnitude for the rib waveguide after the first Si-As_2S_3 transition at $\Delta t \approx 0.15$ indicates that the rib waveguide design results in a lower back-reflection from the Si-As_2S_3 interface, as a result of scattering into the As_2S_3 slab, as shown in Fig. S6.

We fabricated a standalone As_2S_3 chip with identical waveguide cross-sectional geometry and spiral designs used in the Si-As_2S_3 platform. This chip was fabricated using the same process flow as the heterogeneous Si-As_2S_3 platform, with one exception being that the standalone Si-As_2S_3 chip was not exposed to HF before As_2S_3 deposition. The OFDR results from the standalone As_2S_3 chip are presented in Fig. S3. The measured propagation loss for the fully-etched (yellow trace) and 50% etch (purple trace) waveguides were 0.46 dB/cm and 0.40 dB/cm, respectively. These values are much lower than we observed on the Si-As_2S_3 chip and are consistent with previously measured standalone As_2S_3 waveguide losses that are widely reported in the literature, ranging from 0.26-0.5

dB/cm [S1, S2].

Furthermore, the lack of peaks in the 50% etched waveguide on the standalone chip indicates that there is minimal reflection or mode conversion in the waveguide bends. This confirms that the peaks observed in the 50% etched waveguide in the Si-As_2S_3 chip are caused by post-processing and not by the waveguide bend design.

Because the propagation losses on the standalone chip are much lower than measured on the Si-As_2S_3 chip, as summarised in Fig. S4, we conclude that the source of increased losses is due to the post-processing steps used to fabricate the As_2S_3 waveguides and are not fundamental to the waveguide or spiral design itself. The average propagation loss for variants of fully-etched and rib waveguides on the Si-As_2S_3 and standalone chips are annotated in Fig. S4.

Comparing the losses on the Si-As_2S_3 and standalone As_2S_3 chips, we find that there are multiple sources that could contribute to the increased As_2S_3 propagation losses on the Si-As_2S_3 platform. One source of increased loss includes surface roughness induced in the SiO_2 substrate from the HF etching step. We measured the SiO_2 substrate roughness on which the As_2S_3 film was deposited using atomic force microscopy (AFM). We measured a root mean square (RMS) roughness of 0.3 nm before the HF etch step and an RMS roughness of 3.2 nm after HF etching. This could explain why the measured propagation losses for both waveguide designs were higher on the Si-As_2S_3 chip, which was exposed to HF, compared to the overall lower propagation losses on the standalone As_2S_3 platform, which was not exposed to HF. Moreover, the disproportionately larger losses of the fully-etched waveguides on the Si-As_2S_3 chip suggest that the sidewall roughness of the As_2S_3 waveguides on the Si-As_2S_3 platform is higher than the standalone As_2S_3 platform. This can be explained by the rough SiO_2 substrate on the Si-As_2S_3 platform causing roughness on the As_2S_3 film after deposition, which is translated to increased sidewall roughness after etching.

We controlled the HF etch depth by performing multiple etching steps and inspected the etch depth after each step using EBL. Despite this, the HF etching rate was not uniform across the entire trench region. This could lead to localised variations of the As_2S_3 propagation losses and localised scattering, which could explain the peaks in the measured OFDR trace in Fig. S2.

Further investigation is required to fully characterise the sources and relative contributions of the As_2S_3 losses. However, exploring back-side release methods, as demonstrated by Menezo *et al.* [S3] and Mai *et al.* [S4], could provide a path to reducing As_2S_3 waveguides losses on this active Si-As_2S_3 platform.

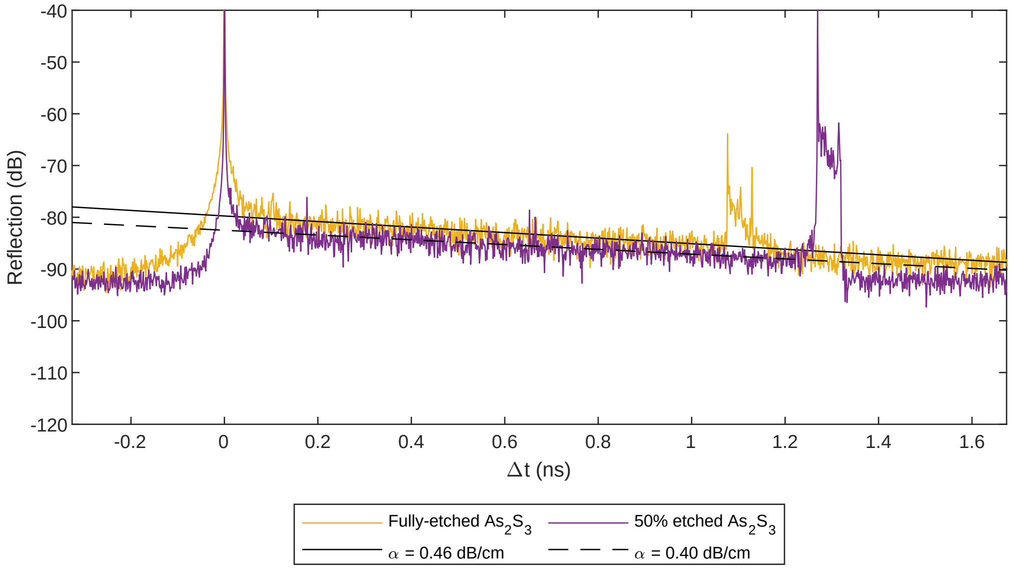

FIG. S3. **OFDR measurement made on the standalone As_2S_3 platform.** The fully-etched (yellow) and 50% etched (purple) waveguides losses are shown. The propagation losses are extracted from the linear fits.

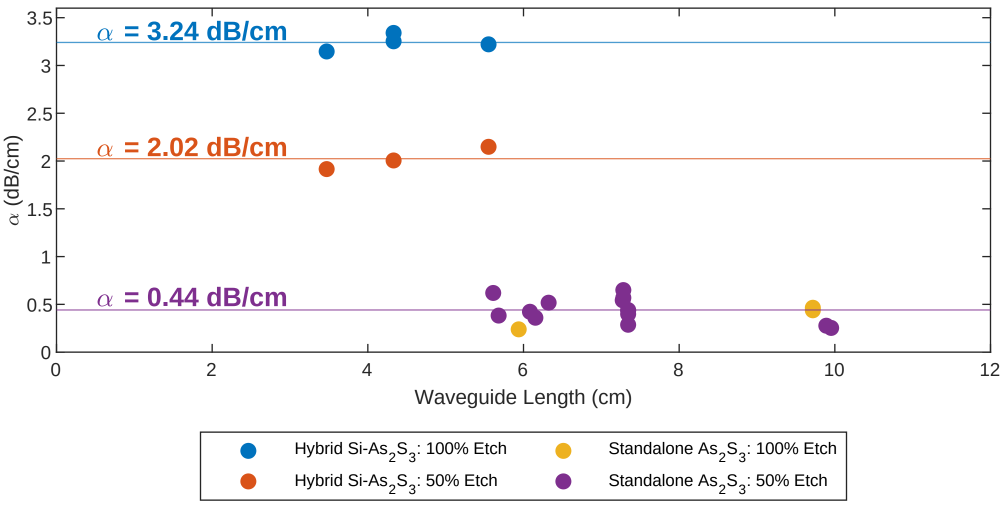

FIG. S4. **Summary of measured As_2S_3 propagation losses.** The measured propagation losses of As_2S_3 waveguides on (blue) fully-etched As_2S_3 on the Si-As_2S_3 platform, (orange) 50% etched As_2S_3 on the Si-As_2S_3 platform, (yellow) fully-etched waveguides on the standalone As_2S_3 platform and (purple) 50% etched waveguides on a standalone As_2S_3 platform. Circles represent the measured propagation loss of each individual waveguide. Lines represent the average propagation loss for each combination of device and As_2S_3 etch.

B. Si-As_2S_3 Interface Loss Calculation

Modifying eq. S1, we can calculate the loss per Si-As_2S_3 interface as

$$L_{\text{Si-As}_2\text{S}_3} = \frac{1}{2}\left(L_\Sigma - \alpha l_{\text{As}_2\text{S}_3}\right) \tag{S2}$$

Using the total losses measured through the Si-As_2S_3 platform, as well as the propagation losses from the OFDR measurements and compensating for the measured losses of the silicon grating couplers, we calculate the loss per Si-As_2S_3 taper and plot the results for the As_2S_3 rib waveguide design with 1800 nm width, 680 nm thickness and 340 nm etch depth in Fig. S5.

The calculated Si-As_2S_3 interface losses have considerable variability between 1.8 and 5.5 dB. If we remove the outlier with the largest loss, we calculate an average loss per Si-As_2S_3 interface of 2.1 dB. A source of variability in the measured Si-As_2S_3 interface originates from the HF etching step. The non-uniformity in the etch depth, discussed above in Section I A, can create abrupt changes in the silicon waveguide, as well as localised over-etching or under-etching, which increases the loss and variability of the Si-As_2S_3 interface. Although there appears to be a trend in the Si-As_2S_3 interface loss as a function of waveguide length in Fig. S5, it is independent of the waveguide length and is significantly affected by the HF etch non-uniformity outlined above. On this platform, the Si-As_2S_3 interface loss measurement is also limited by the number of As_2S_3 spiral waveguides that can be implemented on the chip, as the design rules of the multi-project wafer service limited the on-chip trench size. The specific chip we designed in this work was limited to three As_2S_3 spirals. With future design improvements, more As_2S_3 spirals can be implemented to improve insights regarding the Si-As_2S_3 interface loss.

We used Lumerical eigenmode expansion (EME) to simulate the performance of the Si-As_2S_3 interface, which indicated a loss of 0.39 dB for the 50% etch rib waveguide design. The results of the simulation for the rib waveguide are shown in Fig. S6. Fig. S6 (a-d) shows the TE_{00} mode as the silicon waveguide width changes. Fig. S6 (e-f) shows the propagation of the electric field through the taper in the horizontal and vertical planes through the center of the silicon waveguide, which correspond to z = 110 nm and y = 0 planes, respectively. As the silicon width is reduced, the propagating field transitions from the silicon waveguide to the As_2S_3 waveguide. Scattering into the As_2S_3 slab is observed at the Si-As_2S_3 interface (x = 0 μm) and the silicon waveguide tip (x = 100 μm). The scattering can be reduced by using a fully-etched As_2S_3 waveguide to increase the confinement of the optical mode. However, the effect that the scattering has on insertion loss is minimal, as simulations indicate that using a fully-etched As_2S_3 waveguide reduces the loss per Si-As_2S_3 interface by only $\approx$ 0.1 dB.

We present the corresponding simulation results for the fully-etched Si-As_2S_3 taper in Fig. S7. Although the fully-etched design gives slightly lower interface loss, the optical propagation losses for the fully-etched design are much higher than the rib design, as shown in Fig. S4. The higher propagation losses of the fully-etched design gives a net disadvantage considering overall optical losses and effective length for generating Brillouin gain. Based on the factors discussed above, we used the rib waveguide design presented here to minimise total insertion loss and maximise the effective length of the Brillouin medium.

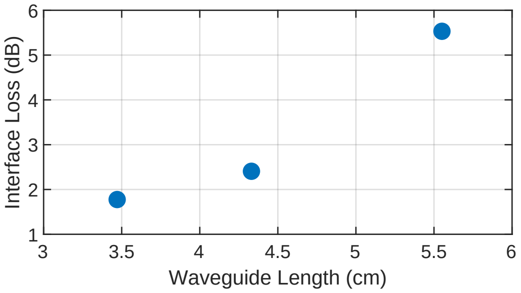

FIG. S5. **Calculated Si-As_2S_3 interface loss.** Each data point represents the calculated insertion loss for the length of As_2S_3 waveguide identified.

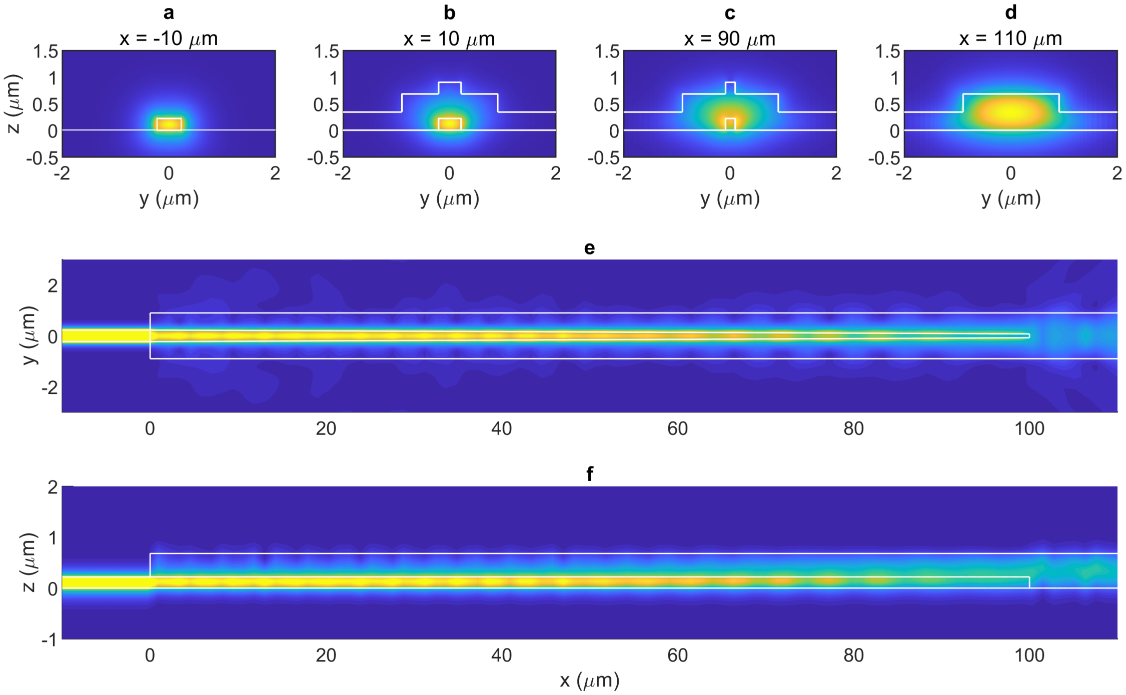

FIG. S6. **Simulation results summary for the Si-As_2S_3 taper with 50% etched As_2S_3 waveguides.** (a-d) TE_{00} modes for several cross-sections through the taper. (e) Propagation of the optical electric field in the plane z = 110 nm. (f) Propagation of the optical electric field in the plane y = 0.

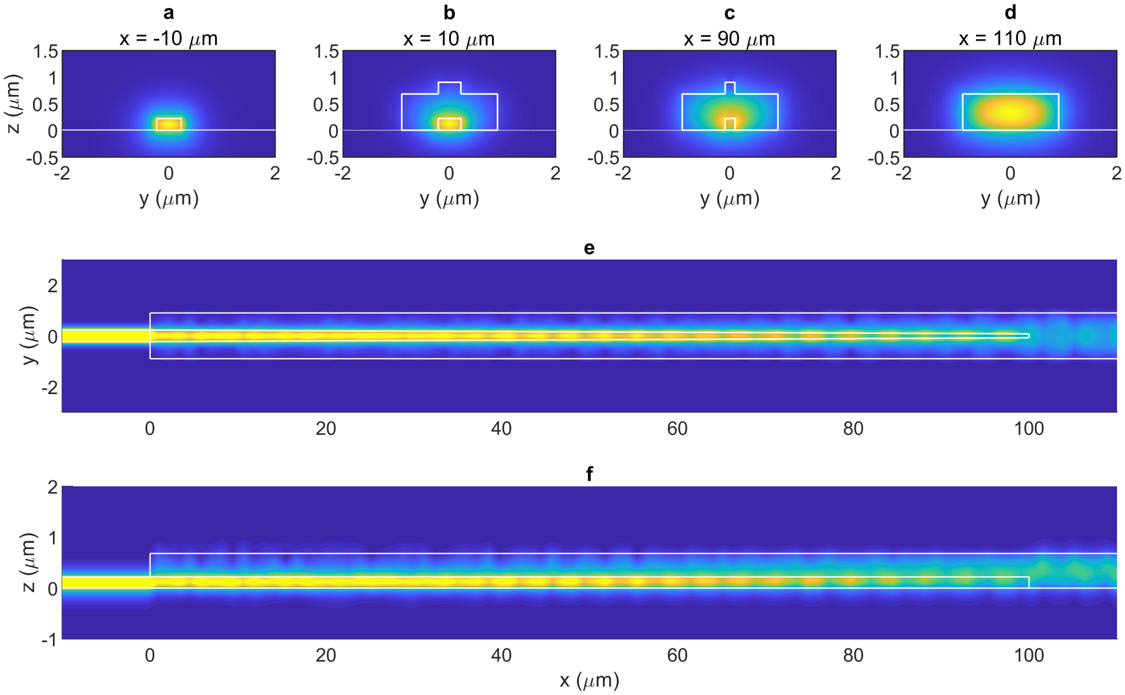

FIG. S7. **Simulation results summary for the Si-As_2S_3 taper with fully-etched As_2S_3 waveguides.** (a-d) TE_{00} modes for several cross-sections through the taper. (e) Propagation of the optical electric field in the plane z = 110 nm. (f) Propagation of the optical electric field in the plane y = 0.

II. ON-CHIP BRILLOUIN GAIN CHARACTERISATION

The SBS gain, G (linear scale), generated in a waveguide with Brillouin gain factor g_{B} (m/W), effective optical mode area A_{eff}, effective length L_{eff} (m) and coupled pump power P (W) is given by [S5]

$$G = e^{g_{\mathrm{B}} L_{\mathrm{eff}} P / A_{\mathrm{eff}}} \tag{S3}$$

The effective length relates to the waveguide length L (m) and propagation loss α by

$$L_{\mathrm{eff}} = \frac{1}{\alpha}\left(1 - e^{-\alpha L}\right) \tag{S4}$$

We note that it is common to report the Brillouin gain coefficient (/m/W) of a waveguide, which is given by $g_{\mathrm{B}}/A_{\mathrm{eff}}$.

A. SBS Gain Measurement

To measure the Brillouin gain response with fine frequency resolution, we use the experimental setup shown in Fig. 5 in the main text. We bias the on-chip DDMZM for single sideband modulation (SSB), and sweep the sideband through the Brillouin gain resonance. Because we use SSB, the measured RF transfer function is proportional to the optical transfer function through the system. To extract the SBS gain response from the underlying MWP link response, we divide the measured response with the pump signal on by the measured response with the pump signal off.

The measured SBS response for pump powers coupled to the As_2S_3 waveguide of 3.2 mW and 17.9 mW is shown in Fig. 3 (d) of the main document. We fit a Lorentzian curve to the measured trace to extract the SBS gain. To observe the Brillouin gain dependency on coupled pump power, we plot the fitted gain as a function of pump power in Fig. 3 (e) of the main document. A linear dependence between SBS gain (dB) and pump power (mW) is observed, as expected from eq. S3. We note that the magnitude of generated Brillouin gain is limited by losses in the As_2S_3 waveguides. The least-squares linear fit gives a Brillouin gain coefficient of 703 /W/m using the measured 3.5 dB grating coupler insertion loss and a Si-As_2S_3 interface loss of 5.5 dB. We note that the Brillouin gain coefficient calculation relies on the measured Si-As_2S_3 interface loss that showed great variation across the measured samples.

B. Increased SBS gain with reduced As_2S_3 losses

We now estimate the expected magnitude of SBS gain with reduced As_2S_3 propagation losses and reduced Si-As_2S_3 interface losses. This models the performance we anticipate when the post-processing of the Si-As_2S_3 chip does not increase the losses of the As_2S_3 waveguides. To calculate the expected magnitude of Brillouin gain, we use the Brillouin gain coefficient that we measured on the Si-As_2S_3 platform and the propagation losses we measured on the standalone As_2S_3 platform. For the coupled power, we used a value of 140 mW, which is 1 dB less than the coupled pump power of 177 mW as recorded by Morrison *et al.* on a passive Si-As_2S_3 platform [S6]. The additional 1 dB loss accounts for on-chip losses from on-chip components required to route the pump in an active Si-As_2S_3 platform. These values are summarised in Table S1. We substitute these values in eq.'s S3-S4 and find an expected Brillouin gain of approximately 18 dB.

TABLE S1. Parameters used to model SBS gain increase.

Parameter	Symbol	Value	Unit	Ref
Brillouin gain coefficient	g_B/A_{eff}	703	/m/W	Measured, This Work.
As_2S_3 propagation losses	α	0.44	dB/cm	Measured, This Work.
As_2S_3 waveguide length	L	5.55	cm	Waveguide design, This Work.
Coupled pump power	P	0.140	W	1 dB less than achieved in [S6]

III. MWP NOTCH FILTER

A. Notch Filter Principle

The RF photonic notch filter principle is outlined in Fig. S8 and relies on two core concepts to achieve large RF notch depths with increased RF link gain. The first concept is destructive interference of RF photocurrents at a given frequency to facilitate a deep RF notch [S7–S9], which is achieved through bias control of the DDMZM and Brillouin processing. The second concept is the control of the CSR to increase the RF link gain. In this section, we outline how these two concepts are implemented using the Si-As_2S_3 platform we present in this work.

The MWP filter is based on the simplified schematic in Fig. S8 (a). The RF input signal, Fig. S8 (b), is modulated onto a continuous wave (CW) laser using a DDMZM, Fig. S8 (c). The CSR is then controlled using on-chip silicon photonic circuitry, as shown in Fig. S8 (d). Brillouin gain is subsequently applied to the upper sideband, shown in Fig. S8 (e), and an off-chip EDFA ensures constant power is maintained at the PD, Fig. S8 (f).

Upon photodetection, Fig. S8 (g), the RF photocurrent consists of two main contributions. One contribution is from the beat between the carrier and upper sideband and another is from the beat between the carrier and lower sideband. For a notch to form, these photocurrent contributions must sum destructively at a given frequency in the RF domain. Therefore, the photocurrent contributions must be equal in magnitude and π out-of-phase. In the passband of the MWP filter, a residual pho-

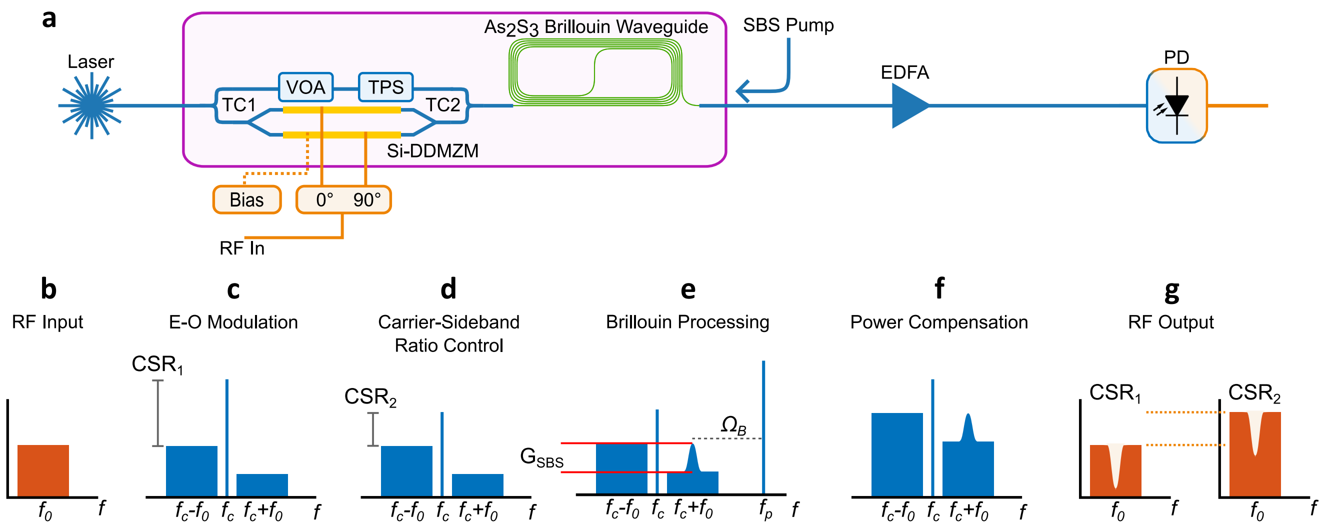

FIG. S8. **Operating principle of the MWP filter.** (a) Simplified schematic. (b) The input RF spectrum is (c) modulated onto a continuous-wave laser using on on-chip DDMZM. (d) In parallel, the CSR is modified to increase the RF link gain. (e) The signal then undergoes Brillouin processing. (f) An off-chip EDFA is used to maintain constant optical power at the PD. (g) Upon photodetection, photocurrent contributions from the upper and lower sidebands destructively interfere at a spectrally-localised point whose frequency offset corresponds to the Brillouin resonance. The RF output is a notch response and the RF link gain of the output is increased through optical CSR control.

tocurrent is formed because the DDMZM bias voltage ensures that the photocurrent contributions are π out-of-phase, but their magnitudes are not equal. However, when the upper sideband coincides with the Brillouin resonant frequency ($\omega_{RF} = 2\pi f_0$), the photocurrent contributions destructively interfere as they are now equal in magnitude, owing to amplification from Brillouin gain. As a result, the photocurrent contributions cancel and an RF notch response is formed, as shown in Fig. S8 (g).

Furthermore, the increase in RF link gain with CSR reduction is exemplified by noting that the RF response with reduced CSR (CSR_2) has larger RF link gain than the RF response with the un-altered CSR (CSR_1), as shown Fig. S8 (g). The notch frequency is controlled by changing the relative frequency offset between the signal laser and the Brillouin pump.

B. Optical Carrier Suppression for RF link gain improvement

The output of a DDMZM driven under small signal conditions by a single RF tone, with angular frequency ω_{RF}, and a $\pi/2$ phase shift between the RF signal in each arm consists of three main components, which may be expressed as

$$E_{-1} = \frac{E_0}{2} J_1(\beta)\left(je^{j\phi_B} + 1\right) e^{j(\omega_c - \omega_{RF})t} \tag{S5}$$

$$E_{C1} = \frac{E_0}{2} J_0(\beta)\left(e^{j\phi_B} + 1\right) e^{j\omega_c t} \tag{S6}$$

$$E_{+1} = \frac{E_0}{2} J_1(\beta)\left(je^{j\phi_B} - 1\right) e^{j(\omega_c + \omega_{RF})t} \tag{S7}$$

where E_0 is the electric field of the signal laser at the input of the DDMZM (V/m), ω_c is the angular frequency of the optical carrier. J_n is the nth order Bessel function of the first kind, β is the modulation index, defined as $\beta = \frac{\pi V_{RF}}{V_{\pi_{RF}}}$, where V_{RF} is the driving voltage of the DDMZM, $V_{\pi_{RF}}$ is the half-wave voltage of the DDMZM, and ϕ_B is the bias phase angle of the DDMZM. $E_{-1,C1,+1}$, correspond to the electric field of the lower sideband, carrier, and upper sideband, respectively.

If this signal is incident on a photodetector, the RF photocurrent at ω_{RF} can be expressed as [S10, S11]

$$i(\omega_{RF}) \propto |E_{C1}||E_{+1}|e^{j(\phi_{+1} - \phi_{C1})} + |E_{C1}||E_{-1}|e^{j(\phi_{C1} - \phi_{-1})} \tag{S8}$$

where $\phi_{-1,C1,1}$ represents the phase of the corresponding optical electric field.

If we define the optical carrier-to-sideband power ratio (CSR) as

$$\mathrm{CSR} = \left(\frac{E_{C1}}{E_{-1}}\right)^2 \tag{S9}$$

Assuming that $E_{-1} \approx E_1$, we can express eq. S8 as

$$i(\omega_{RF}) \propto \frac{E_{C1}}{\sqrt{\mathrm{CSR}}}\left(e^{j(\phi_{+1} - \phi_{C1})} + e^{j(\phi_{C1} - \phi_{-1})}\right) \tag{S10}$$

Expressing eq. (S10) in terms of RF link gain ($s_{21} \propto i^2(\omega_{RF})$) yields the result

$$s_{21} \propto \frac{P_0^2}{\mathrm{CSR}} \tag{S11}$$

Following from eq. S11, we see that the RF link gain is inversely proportional to the CSR. Therefore, we can increase the RF link gain by reducing the CSR if constant power is maintained at the PD.

We implement this concept by using the E-O modulator presented in Fig. 4 (c-d) in the main text and an off-chip EDFA before the PD ensures constant power is maintained at the PD. This is depicted in the high-level schematic in Fig. S8 (a). We show an increase in the RF link gain using the CSR control method outlined in Fig. 5 (e-f) in the main text, which is repeated in Fig. S9 (a-b) for convenience.

We further characterise the trend of increasing RF link gain with decreasing CSR in Fig. S9 (c). A least squares fit reveals a linear dependence between CSR and RF gain, both in decibel scale, with a gradient of -1.05. This is in good agreement with the theoretical model given by eq. (S11), which predicts a gradient of -1. An overall RF link gain increase of 16 dB is observed through CSR control. We note that the reported RF link metrics presented in this work include two RF amplifiers, as shown in the setup in Fig. 5 (a) of the main text, which add a total gain of 47 dB.

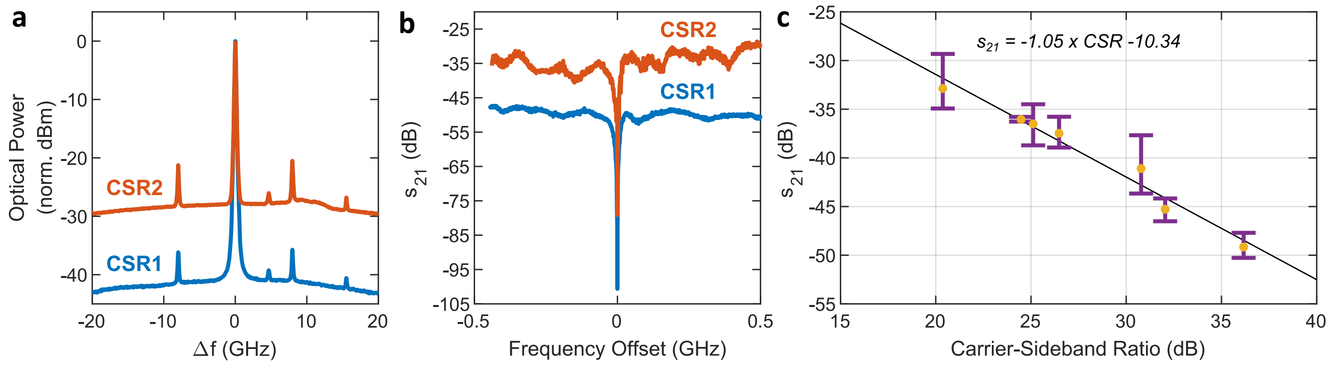

FIG. S9. **RF link improvement using on-chip E-O modulator to control the CSR.** (a) Measured optical spectrum for two different CSRs (CSR1 and CSR2). (b) Measured RF response for CSR1 and CSR2. (c) The measured trend of CSR vs RF link gain. A linear dependency is observed and fit using a least-squares approach.

C. Filter Link Performance Model

We develop the model for determining the RF link performance of the MWP filter we present in this work. The RF link gain of an MWP link using intensity modulation is given by [S12]

$$g_{\text{IM}} = \left(\frac{\pi r_{\text{pd}} R_L P_{\text{opt}} \sin\left(\phi_B\right)}{4 L V_{\pi_{\text{RF}}}} \right)^2 \tag{S12}$$

where r_{PD} is the photodetector responsivity (A/W), R_L is the RF load impedance (typically $50\,\Omega$), ϕ_B is the modulator bias angle, L is the optical loss in the MWP link and $V_{\pi_{RF}}$ is the modulator half-wave voltage.

Given that the MWP filter modulation scheme we present in this work consists of two RF photocurrent contributions, we can express the output power of the MWP link as

$$P = I^2 R = \left(I_{\text{USB}} + I_{\text{LSB}}\right)^2 R \tag{S13}$$

For an MWP link using intensity modulation, the upper and lower sideband photocurrent contributions are equal and in-phase, so we can therefore express the MWP link output power as

$$P_{\mathrm{IM}} = (2I_{\mathrm{LSB}})^2\, R \tag{S14}$$

For the modulation scheme we present in this work, the photocurrent contributions are out-of-phase with each other and differ in magnitude by a factor of $\sqrt{G}$, where G is the magnitude of the applied Brillouin gain. The RF output power of the MWP link can therefore be expressed as

$$P_{\mathrm{DDMZM}} = \left(I_{\mathrm{LSB}} - \frac{I_{\mathrm{LSB}}}{\sqrt{G}}\right)^2 R \tag{S15}$$

By simplifying eq. S15, the MWP link RF output power for the modulation scheme we present in this work can be related in terms of the RF output power for the IM case as follows

$$P_{\mathrm{DDMZM}} = \frac{1}{4}\frac{\left(\sqrt{G}-1\right)^2}{G} \times P_{\mathrm{IM}} \tag{S16}$$

Given that the link gain is proportional to output power, we can therefore express the MWP link gain for the modulation scheme used in terms of the link gain of an IM link using the expression

$$g_{\mathrm{DDMZM}} = \frac{1}{4}\frac{\left(\sqrt{G}-1\right)^2}{G} \times g_{\mathrm{IM}} \tag{S17}$$

We note that in the case of large magnitudes of SBS gain, the modulation scheme used in this work approximates single sideband modulation. As a result, the overall RF link gain is reduced by a factor of 4.

The expression for NF and SFDR can be determined the equations below [S12]

$$NF = P_N - G_{\mathrm{MWP,\ dB}} + 174 \tag{S18}$$

$$SFDR_n = \frac{n-1}{n}\left(IIP_n - NF + 174\right) \tag{S19}$$

$$IIP_3 = \frac{4\left(V_{\pi,\mathrm{RF}}\right)^2}{\pi^2 R} \tag{S20}$$

Where P_N is the RF noise power spectral density, $G_{\mathrm{MWP,\ dB}}$ is the MWP link gain in decibels and 174 represents the noise power spectral density (PSD) of -174 dBm/Hz.

In the following analysis, we assume that the total optical power and therefore the noise PSD remains constant between the DDMZM and IM case.

1. *Performance improvement: Reduced As_2S_3 waveguide losses*

We can calculate the expected improvement in RF link performance with the reduced As_2S_3 waveguide losses using the values presented in Table S1, and the expression for MWP link gain in

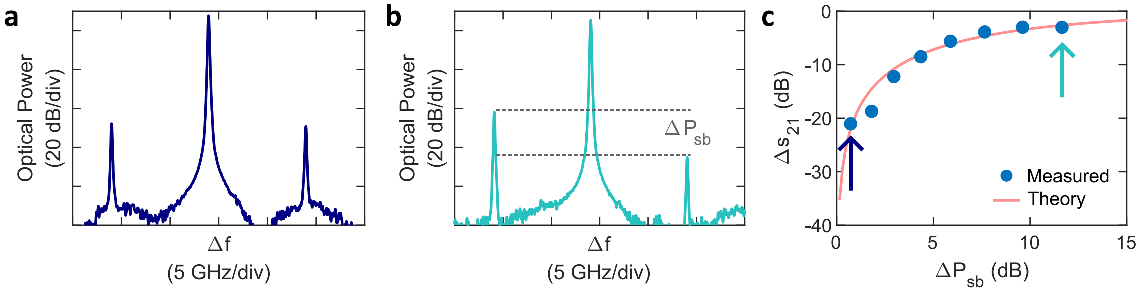

FIG. S10. **Measured MWP link gain as a function of the difference in power between the optical sidebands (ΔP_{SB}).** (a) Measured optical spectrum corresponding to $\Delta P_{\mathrm{SB}} = 0.7$ dB. (b) Measured optical spectrum corresponding to $\Delta P_{\mathrm{SB}} = 11.65$ dB. (c) Measured RF link gain as a function of ΔP_{SB}. The arrows indicate the optical spectra presented in (a-b).

eq. S17. We identified in the Discussion of the main text that reducing the As_2S_3 waveguide losses improved the performance of the MWP filter through two mechanisms.

The first mechanism is from increased optical power at the photodetector. Reducing the loss per Si-As_2S_3 interface from 2.1 dB to 0.1 dB gives a total power increase of 2.0 dB per interface. Reducing the propagation losses from 2.02 dB/cm to 0.44 dB/cm over a 5.55 cm waveguide gives an optical power difference of 8.79 dB. Therefore a total optical power difference of $2 \times 2.0 + 8.79 = 12.79$ dB is to be expected. Because the RF link gain is inversely proportional to the square optical loss in the MWP link, eq. S12, the RF link gain is expected to increase by 25.59 dB from the increase in optical power with reduced As_2S_3 losses.

The second mechanism that improves the RF link performance is from the increased Brillouin gain generated in the As_2S_3 waveguides. This is because the upper sideband amplitude, and therefore the corresponding π out-of-phase photocurrent contribution, is reduced in magnitude as shown in Fig. S8 (e). In section II B, we showed that the generated Brillouin gain is expected to increase from 0.8 dB to 18 dB. Substituting these values into eq. S17, we expect an RF link gain improvement of 19.97 dB due to the increase in SBS gain.

Therefore, with reduced As_2S_3 waveguide losses, we expect a total RF link gain increase of $25.59 + 19.97 = 45.56$ dB. The increased optical power at the PD does not result in any NF and $SFDR_3$ improvement, as the RF noise PSD increases at the same rate as the RF link gain. However, if we consider the effect of increasing the SBS gain on the filter performance, the NF will reduce and the $SFDR_3$ will increase by 19.97 dB. This is because increasing the SBS gain requires the upper and lower sidebands to have different magnitudes, but the total optical power, and hence RF noise PSD, does not change.

We measure the RF link gain as a function of the difference in power between the upper and lower sidebands (ΔP_{sb}) in Fig. S10. We plot the optical spectrum for ΔP_{sb} of 0.7 dB and 11.65 dB in Fig. S10 (a-b), respectively. We plot the measured change in RF link gain (blue circles), Δs_{21}, as a function of ΔP_{sb} in Fig. S10 (c). The measured values are in agreement with the model presented in eq. S17 (solid red line). The arrows indicate the values of ΔP_{sb} presented in Fig. S10 (a-b), highlighting that increasing the difference in power between the optical sidebands, and therefore Brillouin gain, increases the RF link gain.

2. *Performance improvement: As_2S_3 platform with state-of-the-art integrated components*

In this section, we model the best-case MWP link performance of the filter that we present in this work. We do this by substituting reported parameters of state-of-the-art silicon photonic compo-

nents, best-case As_2S_3 losses into eq's. S17, S18, S19. The component values that we used to model the link performance are presented in Table S2.

We plot the MWP link gain, NF, and $SFDR_3$ as a function of SBS gain in Fig. S11 (a-c), respectively. We summarise the MWP link performance for an equivalent laser RIN at the PD of -165 dBc/Hz in Table S3. The results indicate that the link performance deteriorates rapidly for moderate magnitudes of SBS gain (<5 dB), which was a cause of RF performance degradation in this work. Hence, future improvements can be achieved by generating more SBS gain through a reduction in the As_2S_3 waveguide propagation losses.

The performance reported in Table S3 indicates that it is feasible to achieve significant improvements to this platform, which is mainly limited by As_2S_3 losses. For example, with 10 dB of Brillouin gain, it is possible to achieve an MWP link gain of -38 dB, NF of 45 dB and SFDR of 86 dB without RF amplification. This is compared to the RF link gain of -35 dB, a NF of 57.5 dB and $SFDR_3$ of 80 dB that we reported in the main text, which includes two RF amplifiers providing 47 dB of total gain, as shown in the setup in Fig. 5 (a) of the main text.

Still, this performance must be further improved to meet the requirements of future RF systems. To understand the key performance trade-offs for the modulation scheme presented in this work, let us consider the case for small and large magnitudes of SBS gain. For small values of gain, the modulation format resembles PM and the two out-of-phase photocurrent contributions are almost equal in amplitude and therefore leave a small residual photocurrent at the PD output which leads to a reduced RF link gain and SFDR, and an increased NF. As the SBS gain increases, the modulation format resembles SSB modulation and a 6 dB link gain penalty is observed compared to IM, as the net photocurrent is halved because it essentially consists of only a single contribution. Further, the relatively large half-wave voltage of silicon modulators limits the RF link performance.

TABLE S2. Summary of parameters used to simulate MWP link performance on a future Si-As_2S_3 platform

Parameter	Value	Unit	Ref
On-chip Laser Power	115	mW	[S13]
Laser RIN	-165	dBc/Hz	[S13]
Silicon photonic modulator Loss	6.5	dB	[S14–S16]
Silicon photonic modulator $V_{\pi_{RF}}$	6.5	V	[S14–S16]
PD Responsivity	1	A/W	[S17, S18]
As_2S_3 Propagation Losses	0.44	dB/cm	Measured, This Work.
As_2S_3 Interface Loss	0.1	dB	[S6]
As_2S_3 Length	5	cm	N/A
Circulator loss	2	dB	[S19, S20]

TABLE S3. Simulated MWP link performance for different values of SBS gain. The results correspond to RIN = -165 dBc/Hz

SBS Gain (dB)	RF Link Gain (dB)	NF (dB)	SFDR (dB.Hz$^{2/3}$)
1.00 dB	-54.27	61.10	75.49
5.00 dB	-42.17	49.01	83.56
10.00 dB	-38.30	45.13	86.14
30.00 dB	-35.27	42.11	88.16

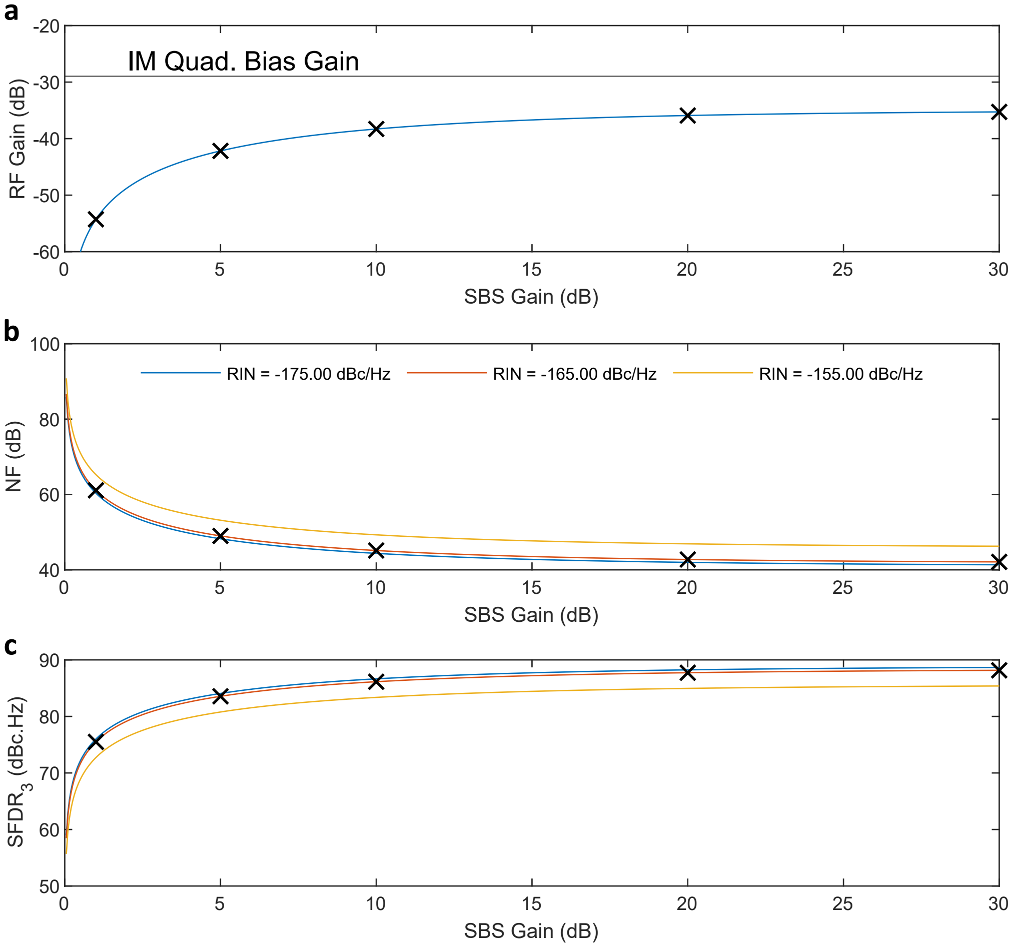

FIG. S11. **Calculated MWP link performance as a function of Brillouin gain.** (a) RF link gain as a function of Brillouin gain. (b) Noise figure as a function of Brillouin gain. (c) Spurious-free dynamic range as a function of Brillouin gain. The crosses represent the values presented in Table S3.

3. RF Notch Depth Sensitivity Analysis

For a notch to form, the resulting photocurrent contributions from each sideband must be equal in amplitude and π out-of-phase. The DDMZM bias voltage changes the amplitude of each sideband. Therefore, changing the DDMZM bias voltage affects the RF notch depth.

To analyse the dependency of the RF notch depth on DDMZM bias voltage, let us consider the spectral components of the DDMZM output under small-signal modulation, given by eq.'s S5-S7. The RF output is formed after photodetection, which is represented by eq. S8.

In the filter scheme presented in this work, the upper sideband is amplified by SBS gain to form the filter notch. The upper sideband after SBS amplification by a factor of G can be expressed as:

$$E_{+1} = \frac{E_0}{2}\sqrt{G}J_1\left(\beta\right)\left(je^{j\phi_B} - 1\right)e^{j(\omega_c+\omega_{RF})t} \tag{S21}$$

In this work, the DDMZM bias was set such that the upper and lower sideband photocurrent contributions were π out of phase and differed in power by the magnitude of applied SBS gain. Assuming 1 dB of applied SBS gain, a DC bias angle of $\phi_B = -0.11$ radians is required. The DDMZM used in this work has a DC half-wave voltage of ≈ 12 V, which corresponds to a voltage of 0.44 V.

Fig. S12 (a) plots the net RF photocurrent outside the Brillouin resonance ($G = 1$) in blue, which corresponds to the filter passband, as a function of DC bias offset from the optimal value of 0.44. Similarly, the net RF photocurrent at the Brillouin resonant frequency (G>1) is in orange, which corresponds to the filter stopband. A significant reduction of the RF output power is observed inside the SBS resonance around $\Delta VDC = 0$ due to the interferometric nature of the MWP notch filtering scheme and corresponds to the RF notch.

Fig. S12 (b) plots the RF rejection ratio as a function of ΔV_{DC}, which is calculated by subtracting the RF passband power from the RF stopband power. Large (>60 dB) RF rejection ratio is observed due to the destructively interfering photocurrents at $\Delta V_{DC} = 0$. The black dashed line marks the RF rejection ratio of 51 dB that we experimentally reported in this work. A rejection ratio of 40 dB can be maintained if $|\Delta V_{DC} < 4.1mV$. The sensitivity of RF rejection ratio on SBS gain is reduced with larger magnitudes of SBS gain, and hence reduced As_2S_3 waveguide losses. For example, with 5 dB of SBS gain, a rejection ratio of 40 dB can be achieved $|\Delta V_{DC} < 14.2mV$.

We note that the sensitivity of RF rejection to bias voltage is not specific to Brillouin-based filters. Many MWP notch filters that use interferometric techniques to enhance RF notch depth have similar sensitivity of RF notch depth to the modulator DC bias voltage.

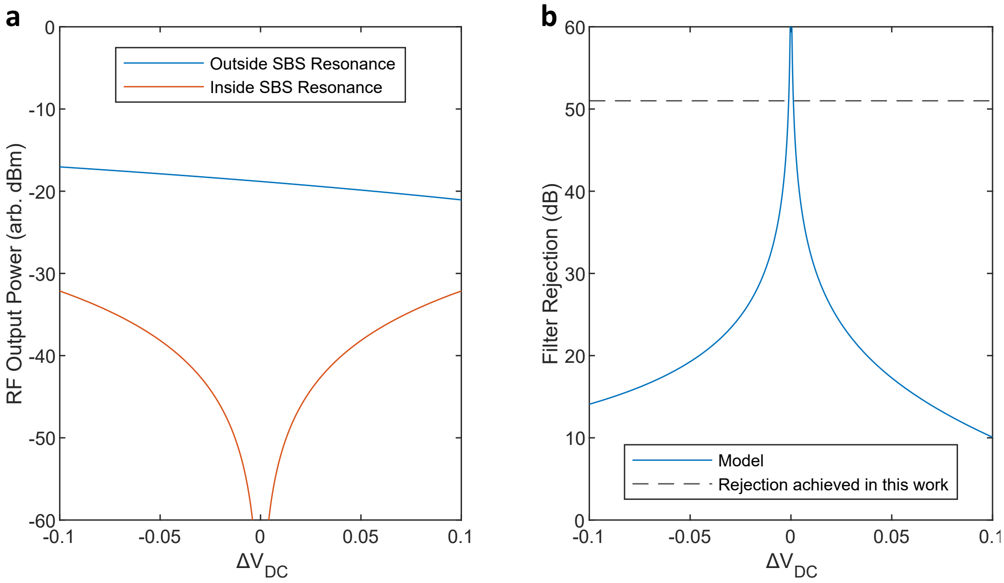

FIG. S12. **Simulated RF rejection ratio dependency as a function of DDMZM DC bias voltage.** (a) Simulated RF output of MWP filter as a function of DC bias voltage for frequencies that are outside the SBS resonance (blue) and frequencies that correspond to the SBS central frequency (orange). (b) RF Rejection ratio as a function of DC bias voltage.

SUPPLEMENTARY REFERENCES

[S1] T. Han, S. Madden, D. Bulla, and B. Luther-Davies, "Low loss Chalcogenide glass waveguides by thermal nano-imprint lithography," *Optics Express*, vol. 18, no. 18, pp. 19 286–19 291, Aug. 2010.

[S2] A. Choudhary, B. Morrison, I. Aryanfar, S. Shahnia, M. Pagani, Y. Liu, K. Vu, S. Madden, D. Marpaung, and B. J. Eggleton, "Advanced Integrated Microwave Signal Processing with Giant On-Chip Brillouin Gain," *Journal of Lightwave Technology*, vol. 35, no. 4, pp. 846–854, 2017.

[S3] S. Menezo, T. Thiessen, G. El-Zammar, J. Mak, J. Da Fonseca, P. Brianceau, B. Szelag, C. Jany, and J. K. S. Poon,

"Back-Side-On-BOX heterogeneous laser integration for fully integrated photonic circuits on silicon," in *45th European Conference on Optical Communication (ECOC 2019)*, Sep. 2019, pp. 1–3.

[S4] C. Mai, P. Steglich, M. Fraschke, and A. Mai, "Back-Side Release of Slot Waveguides for the Integration of Functional Materials in a Silicon Photonic Technology With a Full BEOL," *IEEE Transactions on Components, Packaging and Manufacturing Technology*, vol. 10, no. 9, pp. 1569–1574, Sep. 2020.

[S5] B. J. Eggleton, C. G. Poulton, and R. Pant, "Inducing and harnessing stimulated Brillouin scattering in photonic integrated circuits," *Advances in Optics and Photonics*, vol. 5, no. 4, p. 536, Dec. 2013.

[S6] B. Morrison, A. Casas-Bedoya, G. Ren, K. Vu, Y. Liu, A. Zarifi, T. G. Nguyen, D.-Y. Choi, D. Marpaung, S. J. Madden, A. Mitchell, and B. J. Eggleton, "Compact Brillouin devices through hybrid integration on silicon," *Optica*, vol. 4, no. 8, p. 847, 2017.

[S7] O. Daulay, G. Liu, K. Ye, R. Botter, Y. Klaver, Q. Tan, H. Yu, M. Hoekman, E. Klein, C. Roeloffzen, Y. Liu, and D. Marpaung, "Ultrahigh dynamic range and low noise figure programmable integrated microwave photonic filter," *Nature Communications*, vol. 13, no. 1, p. 7798, Dec. 2022.

[S8] M. Garrett, Y. Liu, P. Ma, D.-Y. Choi, S. J. Madden, and B. J. Eggleton, "Low-RF-loss and large-rejection reconfigurable Brillouin-based RF photonic bandpass filter," *Optics Letters*, vol. 45, no. 13, p. 3705, 2020.

[S9] X. Han, E. Xu, and J. Yao, "Tunable single bandpass microwave photonic filter with an improved dynamic range," *IEEE Photonics Technology Letters*, vol. 28, no. 1, pp. 11–14, 2015.

[S10] P. Zheng, H. Hong, J. Li, G. Hu, B. Yun, and Y. Cui, "Performances of Microwave Photonic Notch Filter Based on Microring Resonator with Dual-Drive Modulator," *IEEE Photonics Journal*, vol. 11, no. 1, pp. 1–13, 2019.

[S11] W. Zhang and R. A. Minasian, "Switchable and tunable microwave photonic Brillouin-based filter," *IEEE Photonics Journal*, vol. 4, no. 5, pp. 1443–1455, 2012.

[S12] D. Marpaung, "High dynamic range analog photonic links : Design and implementation," Ph.D. dissertation, University of Twente, Enschede, The Netherlands, Aug. 2009.

[S13] J. P. Epping, A. Leinse, R. Oldenbeuving, I. Visscher, D. H. Geuzebroek, D. Geskus, A. van Rees, K. J. Boller, M. Theurer, M. Möhrle, M. Schell, C. G. Roeloffzen, and R. Heideman, "Hybrid integrated silicon nitride lasers," in *Physics and Simulation of Optoelectronic Devices XXVIII*, vol. 11274, International Society for Optics and Photonics. SPIE, Mar. 2020, p. 112741L.

[S14] A. Rahim, J. Goyvaerts, B. Szelag, J.-m. Fedeli, P. Absil, T. Aalto, M. Harjanne, C. Littlejohns, G. Reed, G. Winzer, S. Lischke, L. Zimmermann, D. Knoll, D. Geuzebroek, A. Leinse, M. Geiselmann, M. Zervas, H. Jans, A. Stassen, C. Domínguez, P. Muñoz, S. Member, D. Domenech, A. L. Giesecke, M. C. Lemme, S. Member, and R. Baets, "Open-Access Silicon Photonics Platforms in Europe," *IEEE Journal of Selected Topics in Quantum Electronics*, vol. 25, no. 5, 2019.

[S15] X. Xiao, H. Xu, X. Li, Z. Li, T. Chu, Y. Yu, and J. Yu, "High-speed, low-loss silicon Mach–Zehnder modulators with doping optimization," *Optics Express*, vol. 21, no. 4, p. 4116, Feb. 2013.

[S16] X. Tu, T.-Y. Liow, J. Song, X. Luo, Q. Fang, M. Yu, and G.-Q. Lo, "50-Gb/s silicon optical modulator with traveling-wave electrodes," *Optics Express*, vol. 21, no. 10, pp. 12 776–12 782, May 2013.

[S17] X. Xie, Q. Zhou, E. Norberg, M. Jacob-Mitos, Y. Chen, A. Ramaswamy, G. Fish, J. E. Bowers, J. Campbell, and A. Beling, "Heterogeneously integrated waveguide-coupled photodiodes on SOI with 12 dBm output power at 40 GHz," in *2015 Optical Fiber Communications Conference and Exhibition (OFC)*, Mar. 2015, pp. 1–3.

[S18] K. Sun and A. Beling, "High-speed photodetectors for microwave photonics," *Applied Sciences (Switzerland)*, vol. 9, no. 4, Feb. 2019.

[S19] H. Tian, J. Liu, A. Siddharth, R. N. Wang, T. Blésin, J. He, T. J. Kippenberg, and S. A. Bhave, "Magnetic-free silicon nitride integrated optical isolator," *Nature Photonics*, vol. 15, no. 11, pp. 828–836, 2021.

[S20] M. Yu, R. Cheng, C. Reimer, L. He, K. Luke, E. Puma, L. Shao, A. Shams-Ansari, X. Ren, H. R. Grant, L. Johansson, M. Zhang, and M. Lončar, "Integrated electro-optic isolator on thin-film lithium niobate," *Nature Photonics*, vol. 17, no. 8, pp. 666–671, Aug. 2023.
